# Foreign Object Detection in Railway Images Based on an Efficient Two-Stage Convolutional Neural Network

**DOI:** 10.1155/2022/3749635

**Published:** 2022-08-28

**Authors:** Weixun Chen, Siming Meng, Yuelong Jiang

**Affiliations:** Information Engineering Institute, Guangzhou Railway Polytechnic, Guangzhou 510430, China

## Abstract

Foreign object intrusion is one of the main causes of train accidents that threaten human life and public property. Thus, the real-time detection of foreign objects intruding on the railway is important to prevent the train from colliding with foreign objects. Currently, the detection of railway foreign objects is mainly performed manually, which is prone to negligence and inefficient. In this study, an efficient two-stage framework is proposed for foreign object detection in railway images. In the first stage, a lightweight railway image classification network is established to classify any input railway images into one of two classes: normal or intruded. To enable real-time and accurate classification, we propose an improved inverted residual unit by introducing two improvements to the original inverted residual unit. First, the selective kernel convolution is used to dynamically select kernel size and learn multiscale features from railway images. Second, we employ a lightweight attention mechanism, called the convolutional block attention module, to exploit both spatial and channel-wise relationships between feature maps. In the second stage of our framework, the intruded image is fed to the foreign object detection network to further detect the location and class of the objects in the image. Experimental results confirm that the performance of our classification network is comparable to the widely used baselines, and it obtains outperforming efficiency. Moreover, the performances of the second-stage object detection are satisfying.

## 1. Introduction

Railway transport is becoming one of the most popular means of transportation worldwide because of its advantages such as faster speed, higher cost-effectiveness, and better customer comfort [1]. Until the middle of 2021, the national rail operation mileage of China had exceeded 146,300 km. Moreover, according to the latest railway network planning in China, this figure will reach 175,000 km in 2025. However, along with the rapid development of railway transportation, concerns about the accompanying safety issues are also raised, which relate to public property and human life.

Foreign object (obstacle) intrusion is one of the main factors threatening railway safety. In the past, railway safety protection facilities mainly protected the railway from foreign objects by setting up protective nets. However, the protective nets have limited protection against foreign objects with greater mobility, such as pedestrians, large animals, and falling rocks caused by landslides. Collisions of trains with such foreign objects occur from time to time, resulting in large economic loss and casualties. Moreover, railways usually cross remote areas, and if train collisions with foreign objects occur in remote uninhabited places, the rescue will be extremely difficult [2]. Thus, the detection of foreign objects intruding on the railway line is of great significance.

At present, foreign object detection is mainly performed by traditional manual methods [3], which are labor-intensive and time-consuming. Besides, due to the low efficiency of manual methods, it often occurs that foreign objects, such as pedestrians, move to other areas before they are detected. Although the implementation of video monitoring systems greatly improves the detection range and speed, it requires observers to focus on the monitoring video at all times. In particular, to take timely measures to avoid accidents when the train is running at high speed, the system should respond to foreign object intrusion in real time. However, it is infeasible to achieve reliable real-time and 24-hour monitoring via manual methods. Hence, it is significant to automatically detect foreign objects by monitoring video in real time. Early methods typically employ traditional computer vision algorithms [4, 5] or machine learning techniques [6] to detect foreign objects in railway videos or images. However, these methods failed to balance efficiency and accuracy. In other words, they either suffered from low efficiency or failed to meet the efficiency requirements of the foreign object detection systems or poor robustness when faced with various weather and light conditions. Therefore, research into novel real-time computer vision-based foreign object detection methods that can compensate for the shortcomings of traditional methods is demanded for the safety of railways. In this study, we focus on automatically and efficiently detecting foreign objects in railway images captured by a video monitor.

Deep learning-based methods have demonstrated their superiority in various computer vision tasks in recent years, leading to breakthroughs in various fields, including object detection [7], image classification [8], and image segmentation [9].

By employing deep learning techniques, for example, convolutional neural networks (CNNs), it is possible to develop an efficient and effective foreign object detection system for railways. Early attempts have been made to use CNNs for foreign object detection in railway images [10]. However, the efficiency and accuracy of these methods still have room for improvement.

In this work, a two-stage framework is proposed for efficient foreign object detection in railway images. In the first stage, a railway image classification network is proposed to classify any input railway images into one of two classes: normal and intruded. The “normal” class means the railway image is normal without intrusion, and the “intruded” class means there are foreign objects in the images. Then, if the image is classified into the “intruded” class, it will be fed to the second-stage foreign object detection network to further detect the location and class of the objects in the image. The motivation for setting up the first-stage classification network is to improve the efficiency of the foreign object detection system. In particular, by introducing a lightweight classification network in the first stage, the foreign object detection system can scan the railway scenery at a relatively high speed. The second-stage network that has a slower speed can be called only when objects appear in the image. This way, the overall efficiency of the system is increased, because, in most of the cases, there is no foreign object in the images.

**Figure 1:**
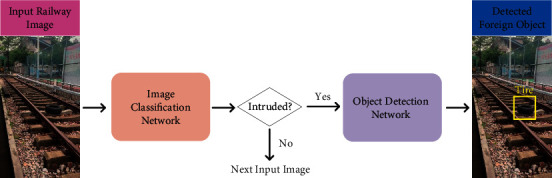
Workflow of the proposed foreign object detection method.

To build the first-stage lightweight classification network, we propose an improved inverted residual unit by implementing the selective kernel convolution [11] and the convolutional block attention module (CBAM) [12] to the inverted residual unit [13]. In the second stage, we directly employ the You Only Look Once v3 (YOLOv3) [14] as our object detector. In Figure 1, we show the overall workflow of our proposed method.

## 2. Related Works

The recent works of foreign object detection methods for railways are introduced in this section.

### 2.1. Traditional Computer Vision and Machine Learning-Based Methods

Salman *et al.* [15] proposed a video analysis-based method for railway foreign object detection. Firstly, the system extracts the target area through optical flow segmentation to detect moving objects. Then, based on the center of the rectangular box corresponding to the object, the ideal trajectory of the center of the object is estimated by designing an implicit Markov model. Ruder et al. [16] proposed a method for obstacle detection in front of the train using edge and texture characteristics of images and optical flow method. Then, they used the Kalman filter [17] to track the targets in images. Oh et al. [18] proposed a passenger safety monitoring system for railroad stations. Through an information fusion module, the video input by the camera is analyzed, and the position of the foreign objects within the railway is located by a stereo vision algorithm. Teng et al. [1] proposed a superpixel-based method for railway foreign object detection, which combines a term frequency-inverse document frequency such as transform and machine learning techniques. Mukojima et al. [19] proposed background subtraction for foreign object detection on railway tracks.

Most of the aforementioned methods typically employed handcrafted features or traditional machine learning techniques. Thus, the applicability and robustness of these methods may fail to overcome the challenges of complicated and various railway scenarios. Recently, convolutional neural networks (CNN)-based methods have shown outstanding performances than traditional computer vision-based methods in a variety of fields. CNNs can learn semantic features from sufficient training data, which get rid of the trouble of traditional manual feature construction. In the following, we introduce the recent progresses of CNNs for foreign object detection methods in railways.

### 2.2. Deep Learning-Based Methods

Ye et al. [20] proposed an end-to-end object detection network, DFF-Net, to detect foreign objects in railways, achieving trade-off between efficiency and accuracy. Cong et al. [21] employed the YOLOv3 [14] model to detect foreign objects on railway tracks. Wang et al. [22] proposed a CNN for foreign object detection by employing residual neural network as main network and a part of single-shot multi-box detection [23] for feature fusion. For vehicle intrusion detection in railway images, Aminmansour et al. [24] designed a region proposal algorithm for generating patches from images and used a CNN to classify the patches. Ye et al. [25] detect foreign objects in front of the train in shunting mode via a feature.

Fusion refine neural network (FR-Net) employed the depthwise-pointwise convolution.

Rail area detection is an important part of a foreign object detection system. For this purpose, Wang et al. [26] proposed a CNN architecture to classify the rail area in pixel-level accuracy, followed by a polygon fitting-based contour optimization method to refine detection results. Wang et al. [27] designed an algorithm for the detection of foreign object intrusion based on adaptive track segmentation. A CNN model was built to classify the track area and the other areas. To overcome the lack of intruding foreign object training samples and provide sufficient training data to the deep learning-based railway foreign object detection methods, Guo et al. [28] developed a C-DCGAN to generate railway images with foreign object intrusion.

Though there are many CNN-based methods for real-time foreign object detection in railway images, the efficiency and accuracy of the existing methods are still required to be further improved to meet the needs of safe railway operation.

## 3. Lightweight Railway Image Classification Network

**Figure 2:**
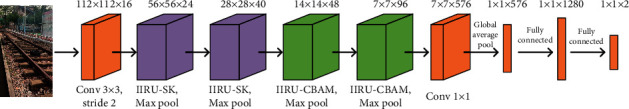
Overall architecture of the proposed railway image classification network. Each rectangle block represents a layer. The operations below the blocks are operations conducted on that layer, and the figures on top of the blocks are the output feature size of that layer. The IIRU-SK is the improved inverted residual unit combined with the selective kernel convolution, and IIRU-CBAM is the improved inverted residual unit combined with the convolutional block attention module.

To increase the efficiency of our method and achieve real-time processing, we build a lightweight network for railway image classification. Any input railway image will be classified into normal or intruded classes by the proposed classification network. In Figure 2, we show the overall architecture of our network. To achieve higher efficiency without losing much accuracy, we use a similar concept of inverted residual unit (IRU) [13] and make two improvements to build our improved inverted residual unit (IIRU):

1. We employ the selective kernel convolution [11] in the inverted residual block to dynamically select kernel size and learn multiscale features from railway images, resulting in the IIRU-SK.
2. To further improve the performance of the proposed network, we employ a lightweight attention mechanism, called the convolutional block attention module (CBAM) [12], to exploit both spatial and channel-wise relationships between feature maps, resulting in the IIRU-CBAM.

In the following, we introduce the modules mentioned above and our IIRU.

### 3.1. Inverted Residual Unit

**Figure 3:**
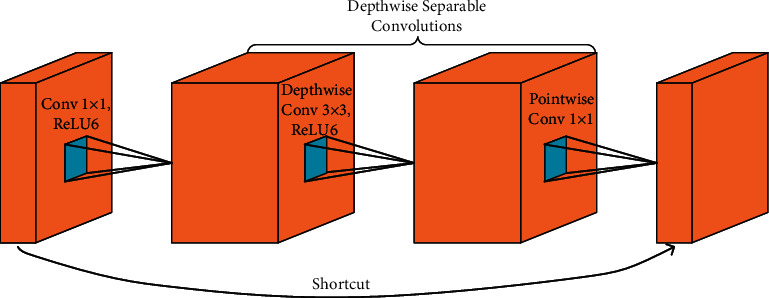
Schematic visualization of the inverted residual unit.

A typical inverted residual unit consists of three convolutional layers and uses depthwise separable convolution [29], as shown in Figure 3.

In the first layer of the inverted residual unit, a convolution kernel of size 1 × 1 is used to expand the input feature maps to a high dimension, followed by ReLU6 as the nonlinearity. Then, the second layer uses a depthwise convolution with kernel size 3 × 3 to further extract features and also uses ReLU6 as the nonlinearity.

Using depthwise convolution, a single convolutional filter is applied to each input channel. This way, computational costs are effectively reduced, and more convolutional kernels can be applied to this layer. Furthermore, in the third layer, a convolution kernel of size 1 × 1 is used to reduce the feature dimension. This step is called pointwise convolution. Finally, the output feature maps of the third convolutional layer are directly fed to the next block, which is called a linear bottleneck. A shortcut connection is built directly between the input and output layers. The depthwise separable convolution is composed of the depthwise convolution and the pointwise convolution.

Given an input tensor *T*_*i*_ of size *h*_*i*_ × *w*_*i*_ × *d*_*i*_ and output a tensor *T*_*o*_ of size *h*_*i*_ × *w*_*i*_ × *d*_*o*_, the computational cost of a depthwise separable convolution with kernel size *k* × *k* is as follows:

(1) $$\begin{array}{c} h_{i}\cdot w_{i}\cdot d_{i}\left(k^{2}+d_{o}\right). \end{array}$$

Regarding traditional *k* × *k* convolution, this cost becomes the following:

(2) $$\begin{array}{c} h_{i}\cdot w_{i}\cdot d_{i}\cdot k^{2}\cdot d_{o}. \end{array}$$

Dividing (1) by (2) yields

(3) $$\begin{array}{c} \frac{h_{i}\cdot w_{i}\cdot d_{i}\left(k^{2}+d_{o}\right)}{h_{i}\cdot w_{i}\cdot d_{i}\cdot k^{2}\cdot d_{o}}=\frac{1}{d_{o}}+\frac{1}{k^{2}}. \end{array}$$

This means that compared with traditional convolutional layers depthwise separable convolution reduces computational costs by about *k*^2^ times. This ensures real-time and accurate railway image classification.

### 3.2. Selective Kernel Convolution

Although the inverted residual unit has high efficiency, it sacrifices some of its accuracies. Thus, to improve the accuracy of our railway image classification network, we improve the inverted residual unit by replacing the depthwise convolution with selective kernel (SK) convolution. Using the SK convolution, the network could capture the spatial information of different receptive field sizes in the railway images. Moreover, the SK convolution is computationally lightweight, which only slightly increases the computational burden and parameters of the network. This property is suitable for the efficiency and accuracy requirements of the railway foreign object detection system. In the following, we introduce the SK convolution detailedly.

**Figure 4:**
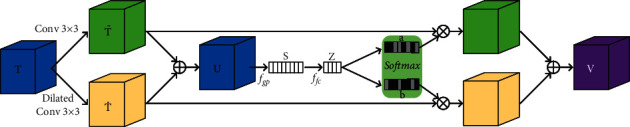
Schematic visualization of the selective kernel convolution.

SK convolution is composed of three main operators: *Split*, *Fuse,* and *Select*, as shown in Figure 4. The *Split* operator generates two paths with different kernel sizes. The *Fuse* operator combines the feature maps produced by the two paths and obtains the global selection weights. Based on the selection weights, the *Select* operator combines the feature maps generated by different paths.

***Split***: for a given input tensor *T* of size *h*′ × *w*′ × *d*′, two branches with convolution kernels of size 3 × 3 and 5 × 5 are used, respectively. Batch normalization and a ReLU nonlinearity are used after the convolutional operations, yielding two distinct feature maps $\tilde{T}$ and $\hat{T}$ of size *h* × *w* × *d*. For efficiency, the convolutional operations are depthwise convolutions and the 5 × 5 convolution is replaced with the 3 × 3 dilated convolution using a dilation size of 2. Although the input tensor can be split into multiple branches, in this work, we only use two branches for efficiency.

***Fuse***: since the two different branches carry information from different receptive fields, an effective fusion operation is required to aggregate the feature maps of the two branches. In particular, $\tilde{T}$ and $\hat{T}$ are first fused through an element-wise summation.

(4) $$\begin{array}{c} U=\tilde{T}+\hat{T}. \end{array}$$

Then, a global average pooling *F*_*gp*_ is applied to produce *s* ∈ *ℝ*^*d*^ that contains channel-wise information extracted by the *Split* operation. In particular, let *s*_*j*_ denote the *j*th element of *s*, and it is calculated as follows:

(5) $$\begin{array}{c} s_{j}=\frac{1}{h\times w}\sum_{m=1}^{h}\sum_{n=1}^{w}U_{j}\left(m,n\right). \end{array}$$

Then, the compact features *z* ∈ *ℝ*^*c*×1^ are generated using a fully connected layer *F*_*fc*_ as follows:

(6) $$\begin{array}{c} z=F_{fc}\left(s\right), \end{array}$$

where *c*=*d*/2. Batch normalization and a ReLU nonlinearity are used after *F*_*fc*_.

***Select***: the compact features *z* are then used to guide the information selection process. In particular, to choose information from different branches, a softmax attention is used to obtain the channel-wise weight vectors as follows:

(7) $$\begin{array}{c} a_{l}=\frac{e^{A_{l}z}}{e^{A_{l}z}+e^{B_{l}z}},\\ b_{l}=\frac{e^{B_{l}z}}{e^{A_{l}z}+e^{B_{l}z}}, \end{array}$$

where *A*, *B* ∈ *ℝ*^*d*×*c*^ are trainable parameters and *a*, *b* are the weight vectors for $\tilde{T}$ and $\hat{T}$, respectively. *A*_*l*_ is the *l*th row of *A* and *a*_*l*_ is the *l*th element of *a*, likewise *B*_*l*_ and *b*_*l*_. Finally, the output feature map *V* is calculated by the weighted sum of the feature maps from the two branches as follows:

(8) $$\begin{array}{c} V_{l}=a_{l}\cdot {\tilde{T}}_{l}+b_{l}\cdot {\hat{T}}_{l},\;a_{l}+b_{l}=1, \end{array}$$

where *V*=[*V*_1_, *V*_2_,…, *V*_*d*_] and *V*_*l*_ ∈ *ℝ*^*h*×*w*^.

### 3.3. Convolutional Block Attention Module (CBAM)

**Figure 5:**
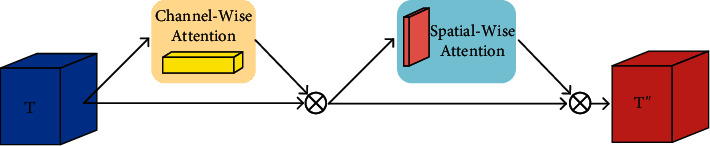
Schematic visualization of the CBAM.

Although there are more advanced attention mechanisms, such as the transformer [30], the computational costs of them are too high to fulfill the real-time requirements of the foreign object detection system. Thus, we employ CBAM, a lightweight weight attention module, to improve the performance of our classification network. The input feature maps are adaptively refined by the channel-wise and spatial-wise attention maps through multiplication operation, as shown in Figure 5.

In particular, for a given input tensor *T* of size *h*′ × *w*′ × *d*′, CBAM firstly generates a 1D channel-wise attention map *M*_*c*_ ∈ *ℝ*^1×1×*d*′^ and then produces a 2D spatial-wise attention map *M*_*s*_ ∈ *ℝ*^*h*′×*w*′×1^. The feature refinement process can be summarized as follows:

(9) $$\begin{array}{c} T^{\prime }=M_{c}\left(T\right)⊗T,\\ T^{\prime \prime }=M_{s}\left(T^{\prime }\right)⊗T^{\prime }, \end{array}$$

where ⊗ is element-wise multiplication and *T*^″^ is the final refined output feature maps.

***Channel-Wise Attention***: in this module, the input tensor or feature map *T* is firstly processed by both average- and max-pooling operations across spatial dimension, resulting in two vectors *T*_avg_^*c*^ and *T*_max_^*c*^, respectively. Then, the channel-wise attention map *M*_*c*_ is generated by feeding the two vectors to a shared multilayer perceptron (MLP) with one hidden layer. For efficiency, the hidden activation size is reduced by a factor *r*. The two output feature vectors of MLP are then merged via element-wise summation. To conclude, the channel-wise attention can be described as follows:

(10) $$\begin{array}{c} M_{c}\left(T\right)=\sigma \left(\text{MLP}\left(T_{\text{avg}}^{c}\right)\right)+\sigma \left(\text{MLP}\left(T_{\text{max}}^{c}\right)\right), \end{array}$$

where *σ* is the sigmoid function.

***Spatial-Wise Attention***: given the output feature maps *T*′ from the channel-wise attention, average pooling and max pooling across channel dimension are conducted to generate two 2D maps *T*_avg_^*s*^ and *T*_max_^*s*^. Then, they are concatenated and convolved by a convolution layer of kernel size 7 × 7, generating the 2D spatial-wise attention map. In summary, the spatial-wise attention can be represented as follows:

(11) $$\begin{array}{c} M_{s}\left(T^{\prime }\right)=\sigma \left(f^{7\times 7}\left(\left[T_{\text{avg}}^{s};T_{\text{max}}^{s}\right]\right)\right), \end{array}$$

where *σ* is the sigmoid function and *f*^7×7^ is the 7 × 7 convolutional operation.

### 3.4. Improved Inverted Residual Unit (IIRU)

**Figure 6:**
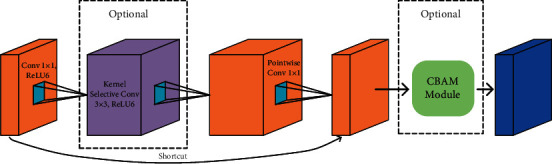
Schematic visualization of the improved inverted residual unit. Compared with the original inverted residual unit, we use SK convolution (purple block) to replace the depthwise convolution. To further improve the performance of the unit, we employ the CBAM mechanism (green box).

In Figure 6, we show the architecture of our proposed IIRU. In particular, we replace the depthwise convolution with the SK convolution, aiming at capturing the spatial information of different receptive field sizes in the railway images. Further, the trainable attention mechanism, CBAM, is implemented after the pointwise convolution layer to perform feature recalibration, which adaptively emphasizes important features and suppresses less useful ones. Note that to reduce computational costs, these two modules are optionally added to the inverted residual unit. Thus, we have two modes of the IIRU, which are IIRU-SK and IIRU-CBAM. IIRU-SK means IRU in combination with the SK convolution, and IIRU-CBAM means CBAM is implemented in IIRU.

## 4. Foreign Object Detection via YOLOv3

After classifying the railway images into normal or intruded classes, we employ the You Only Look Once v3 (YOLOv3) [14] to detect the foreign objects in the intruded images. Different from the two-stage object detection networks [31], YOLOv3 is a one-stage detector. By predicting the locations and classes of the objects in images using one-step regression, the efficiency of YOLOv3 is higher than the two-stage detectors, which is preferable to the foreign object detection system. Moreover, by introducing feature pyramid network (FPN) [32], YOLOv3 is robust to objects at different scales. This makes it suitable to detect foreign objects of different sizes in railway images, such as large objects like vehicles or small objects like boxes. In the following, we briefly introduce YOLOv3.

YOLOv3 uses DarkNet-53 as its backbone feature extractor, which is pretrained on the ImageNet dataset [8]. Its efficiency is about 1.5 to 2 times higher than ResNet-101 and ResNet-152, and the performance is comparable. After the backbone, the concept of FPN is employed to detect targets of different sizes at three different scales. Then, YOLOv3 predicts the locations and classes of the objects by adding several convolutional layers, which output feature maps on three different scales encoding the bounding box, objectness, and class predictions.

Based on the multiscale feature maps, three bounding boxes are predicted by YOLOv3 at each grid cell of the feature maps. Thus, for each scale, the size of the output tensor is *N* × *N* × [3*∗*(4+1+3)], where *N* can be 13, 26, and 52 for the three different scales, and the numbers 3, 4, 1, and 3 correspond to three bounding boxes, four bounding box offsets, one objectness prediction, and three class predictions (there are three classes of objects in our work), respectively.

**Figure 7:**
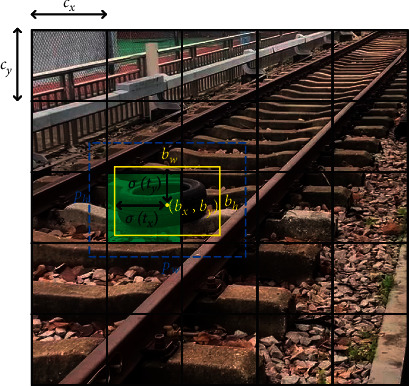
Illustration example of an anchor (blue) and a predicted bounding box (yellow).

To determine the location of the objects, YOLOv3 predicts the center coordinates (*b*_*x*_, *b*_*y*_) of the bounding box with respect to the location of the grid cell. Then, it regresses the width *b*_*w*_ and height *b*_*h*_ of the predicted bounding box from a set of predefined reference boxes called anchors, whose width and height are predefined as *p*_*w*_ and *p*_*h*_, respectively, as shown in Figure 7. Let (*c*_*x*_, *c*_*y*_) denote the cell's offsets from the top left corner of the image, and the predictions can be calculated as follows:

(12) $$\begin{array}{c} b_{x}=\sigma \left(t_{x}\right)+c_{x},\\ b_{y}=\sigma \left(t_{y}\right)+c_{y},\\ b_{w}=p_{w}e^{t_{w}},\\ b_{h}=p_{h}e^{t_{h}}, \end{array}$$

where *t*_*x*_, *t*_*y*_, *t*_*w*_, and *t*_*h*_ are the four predicted offsets of each bounding box and *σ* is the sigmoid function. The objectness score is predicted using logistic regression and the object class is predicted by independent logistic classifiers. More details of YOLOv3 can be found in [14].

## 5. Experiment and Analysis

### 5.1. Dataset

**Figure 8:**
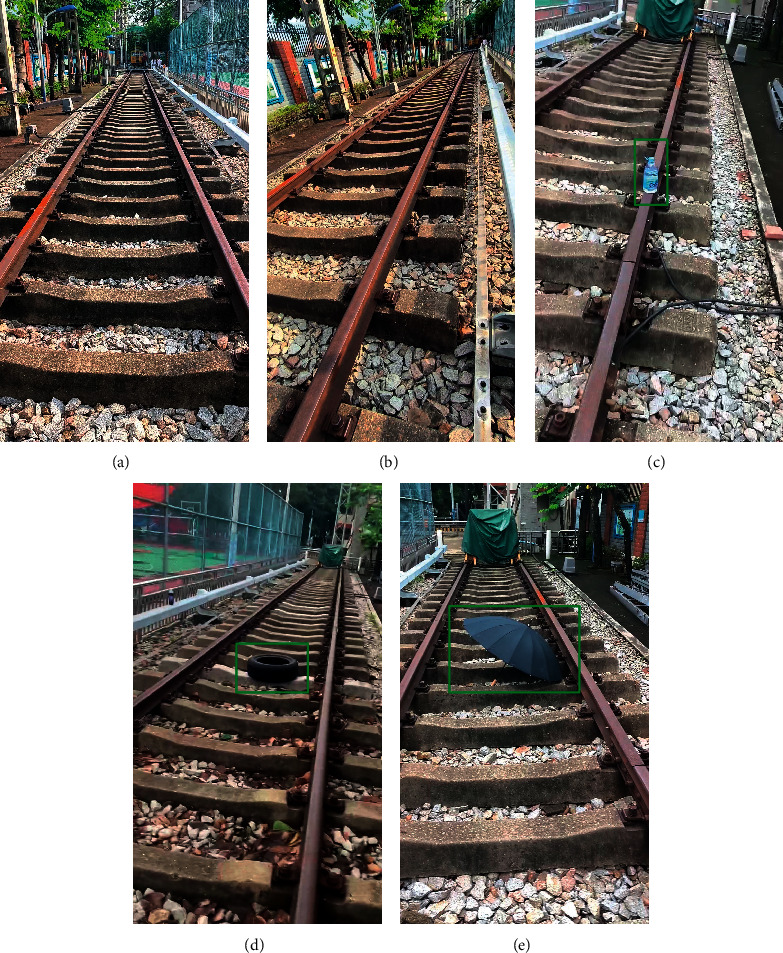
Illustration examples of the railway foreign object dataset. (a, b) Normal railway images. (c–e) Intruded railway images with corresponding reference bounding boxes (green) of the objects.

For the performance evaluation of our proposed method, we built a railway foreign objects dataset including 3145 railway images. It consists of 1523 normal railway images and 1622 intruded railway images. The size of the images is 720✕1280 pixels. The normal railway images were taken from multiple views of a railway scenery that is located at the campus of Guangzhou Railway Polytechnic, Guangzhou, China (Figures 8(a) and 8(b)). To generate the intruded railway images, we placed several foreign objects on the railway and took photographs from multiple views. There are three types of foreign objects in 1622 intruded railway images, including 594 bottles, 712 tires, and 316 umbrellas, as shown in Figures 8(c)–8(e). These objects were selected to simulate possible foreign objects of different sizes intruding on the railway

The reference bounding boxes of the foreign objects were labelled manually.

Among 3145 images, we selected 1885 images to train the image classification network. The remaining 1260 images were used to evaluate their performance in the experiments. In particular, the training set consists of 912 normal images and 973 intruded images, and the test set consists of 611 normal images and 649 intruded images. Then, we used 1622 intruded images to evaluate the second-stage foreign object detection. The remaining 1523 normal images were not used in this stage because there is no object to detect in these images. In particular, 1021, 114, and 487 intruded images were split into training, validation, and testing images, respectively. There are 176 bottles, 204 tires, and 107 umbrellas in 487 test intruded images.

### 5.2. Implementation Details

The railway image classification network was trained using stochastic gradient descent (SGD) optimizer [33]. The momentum was 0.9, the initial learning rate was 0.01, and the weight decay was 0.0005. We set the training batch size to 32. The input images were resized to 224✕224 pixels during training.

We also trained the YOLO v3 using the SGD optimizer with the same settings. The training batch size was set to 16, and the input images were resized to 416✕416 pixels during training.

All the experiments were carried out on a workstation with a single Titan Xp GPU.

### 5.3. Evaluation Metrics

We use precision, recall, and F1 measure to evaluate the accuracy of the image classification network, which are calculated as follows:

(13) $$\begin{array}{c} \text{Precision}=\frac{\text{TP}}{\text{TP}+\text{FP}},\\ \text{Recall}=\frac{\text{TP}}{\text{TP}+\text{FN}},\\ F1-\text{Measure}=\frac{2\text{TP}}{2\text{TP}+\text{FP}+\text{FN}}, \end{array}$$

where TP is true positive, FP is false positive, and FN is false negative.

Moreover, the mean average precision (mAP) [34] was used to evaluate the accuracy of foreign object detection.

Finally, we used frames per second (FPS) to evaluate the efficiency of the compared models.

### 5.4. Experimental Results of Railway Image Classification

In this part, the performances of the railway image classification network are evaluated, in terms of accuracy and efficiency. Three widely used deep learning-based image classification baselines were compared, including vision transformer (ViT) [35], ResNet-50 [36], and MobileNetV2 [13].

In Table 1, we show the comparison results of our proposed railway image classification network with other deep learning-based image classification baselines. We can see that the performances of all the compared models are close to each other. This confirms the performance of our proposed classification network. Although the latest model, ViT, obtained the best performance among all the compared models, its FPS is the lowest and far from reaching the efficiency requirements of the foreign object detection system. Although the mean F1 measure of our model is 0.64% lower than that of ViT, its FPS is the highest and reaches 72.18. Since the FPS of the video produced by the ordinary monitoring system is 60, the FPS of the classification network should exceed 60 so that it can process the video images in real time. From Table 1, we can see that only the efficiency of our proposed model satisfies the real-time requirement of the ordinary video monitoring system, and the other compared models fail to meet this requirement.

Further, from the model size, we can see that our network is very lightweight. The number of parameters is only about 1% of ViT and 40% of MobileNetV2. Thus, our network is much more suitable to implement in a foreign object detection system that has limited computational resources. The experimental results demonstrate that our proposed classification network has higher practical value than the compared methods, because of its relatively high accuracy and outperforming efficiency.

### 5.5. Experimental Results of Foreign Object Detection

**Figure 9:**
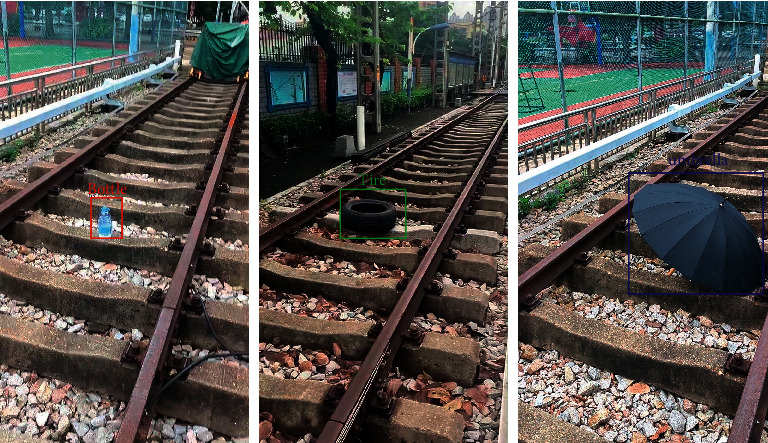
Illustration examples of the detected railway foreign objects.

Here, the foreign object detection accuracy and efficiency of YOLOv3 in the second stage are demonstrated.  Figure 9 illustrates the detection results of the three types of objects. The mAP of YOLOv3 on 487 test images is 85.89%, and the FPS is 32.6. It can be seen that although the accuracy of YOLOv3 is satisfying in our dataset, its efficiency is relatively low. If the foreign object detection system only uses YOLOv3 in all the scenery, it cannot achieve real-time detection. Thus, using our classification network in the first stage, the efficiency of the foreign object detection system is increased.

Further, to simulate the real operation of the foreign object detection system, we test our two-stage framework on all 3145 images in our dataset. In particular, following the workflow illustrated in Figure 1, we feed all these images into our two-stage framework. Then, if there is no foreign object in the current image, the next image will be fed to the framework; otherwise, YOLOv3 will be used to detect foreign objects in the image. Finally, the FPS of our two-stage framework reaches 51.77, which is close to the 60 FPS requirement of the foreign object detection system.

## 6. Conclusion

This study proposes a two-stage framework for efficient foreign object detection in railway images. The first stage is a railway image classification network that classifies any input railway images into one of two classes: normal and intruded. Then, if the image is classified into the “intruded” class, it will be fed to the second-stage foreign object detection network to further detect the location and class of the objects in the image. By setting the first-stage classification network, we increase the efficiency of the foreign object detection system.

To build the first-stage lightweight classification network, we propose an improved inverted residual unit by implementing the selective kernel convolution and the convolutional block attention module (CBAM) to the inverted residual unit. The experimental results show that the practical value of our proposed classification network is higher than that of the compared methods, because of its relatively high accuracy and outperforming efficiency.

## Figures and Tables

**Table 1:** Results of comparison with other deep learning-based classification baselines for railway image classification.

	Mean precision (%)	Mean recall (%)	Mean F1 measure (%)	FPS	Model size (M)
Proposed	96.88	96.82	96.85	72.18	0.90
ViT	97.51	97.48	97.49	38.94	85.80
MobileNetV2	96.25	96.19	96.22	59.58	2.23
ResNet-50	96.66	96.24	96.64	52.23	23.51

## Data Availability

The datasets used and analyzed during this study are available from the corresponding author upon request.
